# Implementation of a Clinical Decision Support Platform for the Efficient Liberation and Assessment of Feeding and Nutritional Data in a Tertiary Neonatal Intensive Care Unit

**DOI:** 10.1016/j.mcpdig.2023.09.002

**Published:** 2023-10-23

**Authors:** Tammi L. Jantzen, David R. Genetti, Aamir A. Nayeem, Ashley S. Ross, Laura E. Carroll, Misty L. Virmani

**Affiliations:** a Astarte Medical, Yardley, Pennsylvania; b Department of Pediatrics, University of Arkansas for Medical Sciences, Little Rock, Arkansas

## Abstract

Hospitals collect vast amounts of discrete patient data points, such as diagnoses, demographic characteristics, growth measures, and laboratory findings, all of which are stored in the electronic medical record (EMR). The early promise of using the EMR to support research and provide clinical decision support often falls short. Clinical practice groups generate a wealth of collected data but have no way to assess the quality of this data let alone mine it for useful insights. The purpose of this work was to provide the neonatal intensive care unit (NICU) clinical team with a digital tool to access feeding and nutrition data for preterm infants. The tool (1) extracts data directly from the EMR; (2) analyzes the data to identify missing, incorrect, and inconsistent entries; (3) structures the data for feeding models targeting nutrition-related outcomes and quality improvement initiatives; and (4) evaluates adherence to the hospital’s consensus-based feeding protocols. The EMR-integrated solution, NICUtrition, was implemented at Arkansas Children’s Hospital and provided longitudinal, high-resolution data related to feeding and nutrition for 1992 patients over a 5-year period. This included over 1.2 million feeding events, each with extensive detail. Enteral feeding protocols used during this time were digitized, and historical protocol adherence was evaluated. Visualizations of this data through NICUtrition provided detailed insights on feeding, growth, milestones, protocol adherence, and effectiveness, along with insights related to charting inconsistencies, missing data points, and inaccurate information in the EMR.

As the survival of extremely preterm infants improves, attention is focused on improving the quality of survival through optimal nutrition management. Despite accumulating research and increased awareness of preterm infant nutritional requirements, optimal feeding practices and nutritional management have been constrained by a lack of resources, shared knowledge, and time. Nutritional shortcomings do not often require urgent intervention during periods of critical illness; however, failure to identify early deficits increases rates of faltering growth and poor cognitive outcomes. Implementing and adhering to a set of standardized feeding guidelines has been shown to improve outcomes and decrease co-morbidities.^1^

However, the ability to monitor adherence to consensus-based feeding protocols or to gather useful insights from feeding data is absent from current work environments. The issue is not lack of available data but rather lack of ability to capture and transform this data from the electronic medical record (EMR) into actionable insights for clinicians to alter care in real time.

Arkansas Children’s Hospital (ACH) partnered with Astarte Medical to implement NICUtrition, an EMR-integrated digital tool accessing feeding and nutrition data. The purpose of NICUtrition is to track enteral feeding protocol effectiveness and adherence and to predict enteral feeding progression.

Arkansas Children’s Hospital is a 336-bed, Magnet-recognized facility in Little Rock, operating the state’s only Level IV neonatal intensive care unit (NICU) with 104 beds.

NICUtrition has several essential components that enable care teams to optimize and streamline the feeding practices: EMR data liberation through a proprietary Integration Harness, protocol digitization and adherence analysis, and an interface that automates calculations and tracks milestones and outcomes. NICUtrition’s analytics provide unit-wide and population-wide health capabilities allowing teams to assess quality improvements, support training and education and efficiently conduct research to drive new models of care and interventions.

### The Implementation Process

NICUtrition runs as a cloud-hosted application managed by Astarte Medical and built using modern web standards, adhering to an Amazon Web Services well-architected framework. This cloud-based approach enforces application access requirements and configurations, Health Insurance Portability and Accountability Act compliance, and general application security and reliability. It collects patient anthropometrics, medications, diagnoses, nutrition, and feeding data directly from Epic using secure fast healthcare interoperability resources (FHIR) application programming interfaces (APIs), a standard for exchanging patient health information. NICUtrition customer environments run on robust, dedicated, and single-tenant resources.

The first step in the implementation process for the Information Technology team at ACH was a 5-year retrospective extract of NICU data (November 5, 2017 - March 24, 2022). Data for 1992 patients admitted to the NICU by day of life 3 over a 5-year period were extracted for infants born less than 34 weeks gestational age and/or weighing less than 2500 grams. A Business Intelligence Developer (BID) at ACH mapped the required data elements and facilitated the data extract and transfer. These data elements included patient information, fluids, growth measurements, parenteral, gavage and oral nutrition, and neonatal morbidities.

The BID worked closely with an ACH clinical analyst who was familiar with the hospital’s build of Epic and a subject matter expert. The clinical analyst served as the liaison between the BID and the NICU clinical team. The BID estimated 80-100 hours were spent on the retrospective data extract. However, the involvement of multiple parties across several organizations to discover the location of critical data and identify the comprehensive list of flowsheet rows prolonged the implementation process considerably in number of days.

An essential component to the NICUtrition platform is the proprietary Integration Harness. It works in the background (users do not interact with it) by accessing granular, detailed clinical workflow information, validating data quality, combining fields and performing calculations where required, and standardizing this data for ingestion into NICUtrition. At a population health level, the standardization provided by the Integration Harness enables comparison of de-identified data across units, hospitals, and regions regardless of EMR and supports future efforts in machine learning-based predictions and personalization. The challenge was discovering and correcting nuanced variations in the data. In addition, Astarte Medical did not have direct access to the patient records through the Epic interface, so there was no definitive way to know if the data received was correct or comprehensive, without clinical team involvement. Appropriateness criteria were established and enforced. The serial discovery of these variations added time to the implementation process.

The next step after the retrospective data extraction was to digitize the 3 enteral feeding protocols used over the 5-year historical period, each of which includes use of parenteral nutrition, enteral feeding rates, advancements, target fortification (calorie) details, and contraindications. Logical “decision trees” were created to then assess protocol adherence. The Astarte Medical team built these decision trees through an iterative process of review with ACH’s clinical team and validated them against historic data to confirm interpretation and tolerances.

The ACH NICU clinicians viewed the 5-year retrospective data through the NICUtrition dashboard (Figure 1), including a comparison of all 3 protocols and associated adherence correlated to outcomes. This enabled the clinical team to use benchmarking filters (Figure 2) to perform comparative studies and measure the success of different protocols and other quality improvement efforts.

**Figure 1:**
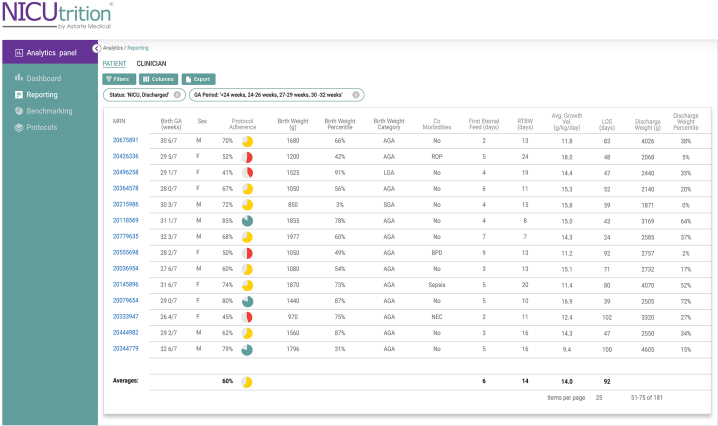
NICUtrition analytics panel reporting screenshot. The reporting screen provides a summary of metrics for a selected cohort to enable visibility into unit-wide averages.

**Figure 2:**
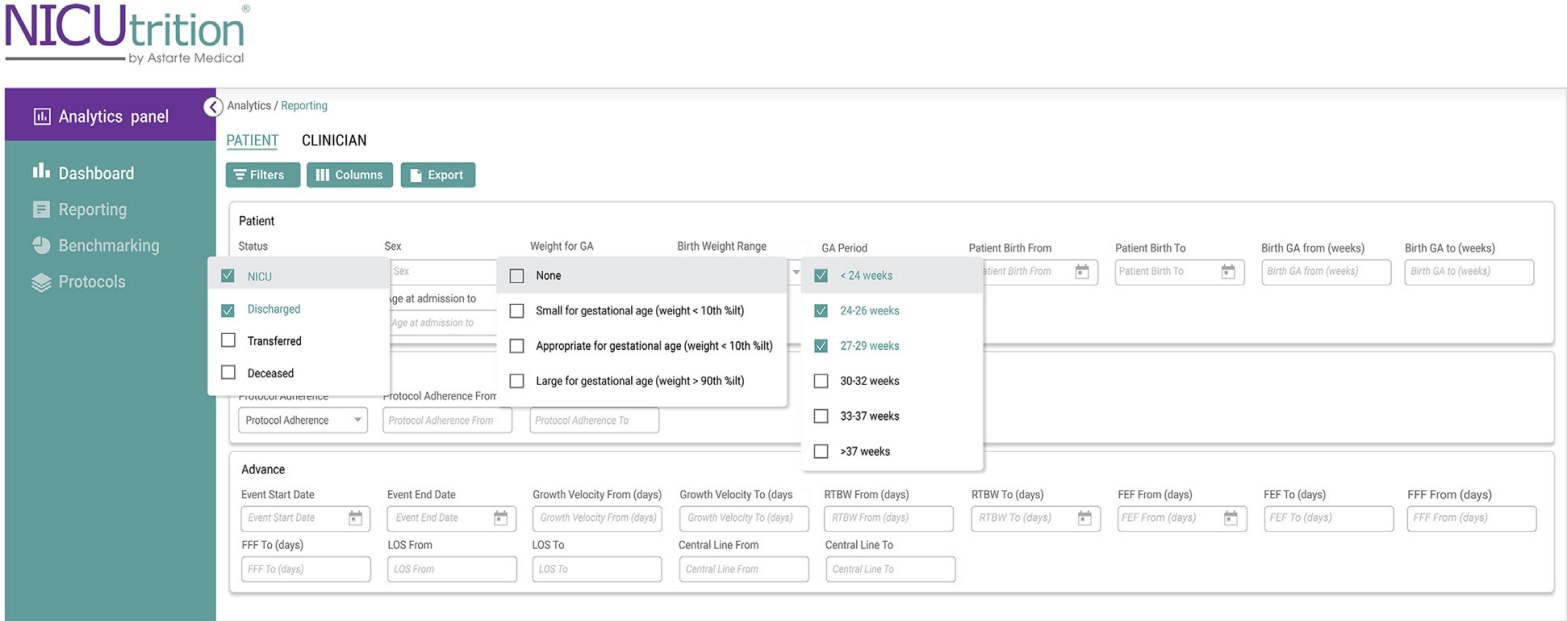
NICUtrition filter and sort functions. Example of the filter and sort functions of the software which allows for the ability to create specific patient cohorts for analysis.

The final step was accessing data through the FHIR APIs for real-time access to prospective patient data. The Astarte Medical team worked directly with ACH’s Information Technology team and a dedicated Epic liaison to facilitate the prospective launch of NICUtrition. Once a clinical user views an individual patient chart, they can launch NICUtrition with full user and patient context from a tab deployed to the Epic interface. Data is regularly synchronized, and can be synchronized on-demand by a user accessing a particular patient through the NICUtrition tab. Because NICUtrition is integrated into Epic, it fits into the existing clinical workflow and does not create the need for additional documentation.

As expected, there were challenges with accessing data in real time. Many of the API end points were being exposed for the first time by ACH. There were technical complications that needed to be navigated, like customized internal redirect rules that were routing the requested traffic in unexpected ways. Many of the FHIR API responses would claim successful execution even when they were explicitly excluding important data. Being among the limited few using FHIR API services, finding the available Information Technology or Epic customer resource to help resolve underlying issues was difficult as they were already fully allocated to other work. Resolving these data access issues required extra team meetings with representatives from the hospital’s network and API teams, Astarte Medical and Epic.

As with all FHIR-based applications, patient enrollment into a clinical decision support (CDS) tool like NICUtrition can be challenging because the FHIR standard is all “opt-in” by design. A clinical user is required to launch the NICUtrition tab from an individual patient chart to enroll an infant. Until that happens, NICUtrition cannot access data on the patient. Including enrollment in the NICU admissions process will solve this problem. Some data are only accessible for 72 hours through the available APIs, so timely enrollment is critical.

The application is limited by the timeliness and quality of the data available. In addition, feeding protocols (or feeding practices) occasionally include observational inputs that are not charted in the EMR and these important inputs are not available for analysis.

### Insights and Unexpected Results

Arkansas Children’s Hospital and Astarte Medical have interrogated the data using the platform and discovered publishable findings which have led to accepted abstracts at several leading conferences, as mentioned below:

(1) The retrospective data review of the high-resolution feeding details revealed 2550 unique feeding order combinations (recipes) with over 1000 of those feeding orders occurring 2 times or less. Given a history of deploying consensus-based protocols to help standardize feeding, this finding was a surprise to the clinical team. After further research, it was discovered that some of these unique order combinations were because of the way people were free texting the feed orders. Other errors in charting were discovered, including inaccurate weights and lengths, such as a birth weight of 3.89 grams and a length of 3.2 cm. On the basis of these insights, the clinical team plans to streamline feeding orders to decrease variability and error, with the goal of decreasing the number of unique feeding orders and improving protocol adherence.^2^
(2) Three feeding protocols with successive implementation were analyzed across 3 cohorts of infants for protocol adherence and outcomes. Comparing protocol 1 with 3 revealed a statistically and clinically significant decrease in days until first fortification in all cohorts (Cohort 1 (<30 weeks gestational age at birth) *P*=.0069; Cohort 2 (30 0/7 - 32 6/7 gestational age at birth) *P*=.0203; Cohort 3 (Very low birthweight <1500 grams) *P*=.0027). Time to full enteral feeding volume decreased in all identified cohorts and adherence to protocols increased steadily from protocol 1 to 3. Having the ability for real-time monitoring of protocol adherence and patient outcomes enables the clinical team to continuously educate staff and make adjustments to improve patient outcomes.^3^

(3) It has been widely published that there is considerable disparity in quality-of-care delivery based on infant race between and within NICUs. Using NICUtrition analytics, unit-wide tracking of key metrics was provided by displaying a side-by-side comparison of nutrition metrics, feeding milestones, growth measures, and discharge statistics by race. Comparing enteral feeding milestones, days to first enteral feed, first fortification, and first full enteral feed were either consistent or faster for Black infants compared with White infants. Adherence to enteral feeding protocols was also relatively consistent for White and Black infants. However, the rate of human milk at discharge was substantially less for Black infants compared with their White counterparts. By using this data, a greater focus can now be placed on improving adherence to breastfeeding at discharge.^4^
(4) Two hundred and seventy-three infants born under 33 weeks gestational age and admitted to ACH by day of life 3 were placed in 3 cohorts on the basis of the total percentage of their enteral diet during their NICU stay that came from human milk (HM). Comparing the high HM diet (cohort 1) with the low HM diet (cohort 3), days on parenteral nutrition, central line days, and length of stay were less in cohort 1 compared with cohort 3. Further investigation on the optimal dose, length of exposure, and source of HM would be beneficial for improving the understanding of preterm infant needs.^5^

### Why Data Quality Matters

The current state of clinical EMR data is not ready for artificial intelligence. To realize the potential of artificial intelligence, clean, accurate, and standardized data are needed to fuel the development of algorithms. NICUtrition identified documentation gaps and areas where data quality was lacking or inconsistent and informed the clinical team of areas for potential process improvements for documentation and ordering. NICUtrition was used for early identification of “user input” errors, such as incorrect weights, lengths, and heights, feeding entries, and fluid details. It provided a feedback loop that identified values needing review. Without this feedback loop, there is little immediate incentive to correct inaccurate data while the accurate information is still available to the providers, becoming increasingly impossible to correct months or years later. Real-time validation and feedback will result in dramatically improved data quality, greater reliability on EMR data, and better data sets for all future data driven efforts, including artificial intelligence.

### Conclusion

Feeding and nutrition is a data-intensive, target-driven effort that can be improved using CDS tools to automate calculations, implement best practices, increase confidence in feeding decisions, and improve data quality and communication among the clinical team. Implementing software that harnesses and transforms EMR data can identify complex clinical patterns and assist clinicians in decision-making, diagnostics, and optimizing feeding and nutrition plans for preterm infants.

## Potential Competing Interests

Tammi L. Jantzen, David R. Genetti, and Aamir A. Nayeem are employees and equity holders of Astarte Medical. Dr Ashley S. Ross, Dr Laura E. Carroll, and Dr Misty L. Virmani have no conflicts of interest to disclose.
